# The effect of red/blue color stimuli on temporal perception under different pupillary responses induced by different equiluminant methods

**DOI:** 10.1371/journal.pone.0270110

**Published:** 2022-06-21

**Authors:** Yuya Kinzuka, Fumiaki Sato, Tetsuto Minami, Shigeki Nakauchi

**Affiliations:** 1 Department of Computer Science and Engineering, Toyohashi University of Technology, Toyohashi, Aichi, Japan; 2 Electronics-Inspired Interdisciplinary Research Institute, Toyohashi University of Technology, Toyohashi, Aichi, Japan; Groningen University, NETHERLANDS

## Abstract

As time plays a fundamental role in our social activities, scholars have studied temporal perception since the earliest days of experimental psychology. Since the 1960s, the ubiquity of color has been driving research on the potential effects of the colors red and blue on temporal perception and on its underlying mechanism. However, the results have been inconsistent, which could be attributed to the difficulty of controlling physical properties such as hue and luminance within and between studies. Therefore, we conducted a two-interval duration-discrimination task to evaluate the perceived duration of color stimuli under different equiluminant conditions: subjective or pupillary light reflex (PLR)-based equiluminance. The results, based on psychometric functional analyses and simultaneous pupillary recordings, showed that the perceived duration of red was overestimated compared with blue even when the intensity of the stimulus was controlled based on subjective equiluminance (Experiment 1). However, since blue is known to induce a larger PLR than red despite equiluminance, we conducted a controlled study to distinguish the indirect effect of pupillary response to temporal perception. Interestingly, the effect observed in Experiment 1 faded when the luminance levels of the two stimuli were matched based on PLR response (Experiment 2). These results indicate that duration judgement can be affected not only by the hue but also by different equiluminance methods. Furthermore, this causality between the equiluminance method and temporal perception can be explained by the fluctuations in incident light entering the pupil.

## Introduction

As color is indispensable to individuals’ perceptual experiences of the world, its effect has been the subject of research since the early history of psychology. Studies show that colors surrounding us in our daily lives profoundly affect bodily functions, including mood and behavior [1, 2]. The potential effect of color on our bodily functions has been extensively discussed after Hill and Barton reported that competitors wearing red clothing or body protection had a significantly higher chance of winning the 2004 Olympic Games in Athens [3]. They explained that this is because the vividness of the red color increases the amount of testosterone they have in their bodies, which also correlates with physical health, leading to a psychological advantage.

Although that study prompted much controversy, people have cognitive associations with specific colors. For instance, long-wavelength colors such as red are often considered dangerous and compelling, whereas short-wavelength colors such as blue are perceived as relaxing. Furthermore, red explicitly captures our attention and is associated with biological danger [4, 5], whereas blue is often considered to evoke the opposite response. Numerous researchers have discussed the psychological effects of colors, with most studies explicitly focused on red and blue, as these are two of the three primary colors located at each end of the visible-light region. Assumptions on red-inducing arousal have also been extensively investigated via physiological measures such as electroencephalography (EEG) [6], galvanic skin response [7], and heart rate variability [8]. Moreover, since color emerges in physiological indices, it is known to affect human behavior, such as cognitive performance. Furthermore, red may enhance task performance, unlike blue [9, 10]. The influence of color on cognition and behavior is typically a learned association, which occurs when different colors are accompanied by specific experiences or concepts (*e*.*g*., red is often associated with danger, such as stop signs and warnings) [11]. Therefore, varied associations involving red and blue can induce alternative bodily functions; red induces arousal, while blue is relaxing.

Unsurprisingly, the growing interest in colors’ psychological effects on cognitive functions includes the pursuit of their effect on temporal perception was no exception. We also know from experience that perceptual time is not isomorphic to physical time, and our subjective experience of the passage of time is significantly influenced by non-temporal aspects, such as emotions, experiences, and our internal states. The fundamental role of time in social activities has led to temporal perception studies since the earliest days of experimental psychology [12, 13]. Psychophysical and behavioral techniques help demonstrate how non-temporal cognitive information modulates the subjective perception of time to reveal the underlying mechanism of temporal perception. Many studies have been conducted using stimuli with different visual sensory inputs to investigate how the apparent duration of a specific time interval is influenced by lower-order characteristics and by color. Since the 1960s, the potential effects of red and blue on temporal perception have been studied to determine the fundamental mechanism. Table 1 summarizes the methods and results of several temporal perception studies concerning color.

**Table 1:** Summaries of studies on the effect of red/blue colors on temporal perception (modified after Yang et al.).

Study	Method		Results (Judged longer)	Statistical values
	Task	Interval duration		
Smets et al. (1969) [14]	Estimation	45 s	Blue > Red	t = 1.55, Cohen’s d = 3.07^†^
Caldwell et al. (1985) [15]	Production	30 s, 40 s	No effect	-
Gorn et al. (2004) [16]	Estimation	17.5 s	Blue < Yellow/Red	Blue-Yellow: F(1, 47) = 3.95
				Blue-Red: F(1, 59) = 5.21
Katsuura et al. (2007) [17]	Production	90 s, 180 s	Blue > Red	Blue: t = 1.05, Cohen’s d = 0.35^†^
				Red: t = 1.00, Cohen’s d = 0.33^†^
Shibasaki et al. (2014) [18]	Comparison	0.4–1.6 s	Blue < Red ^1^	F(1,72) = 14.42, $\eta_{p}^{2}$ = 0.17
Thönes et al. (2018) [19]	Comparison	1.6–2.4 s	Blue > Red	t = 2.917, Cohen’s d = 0.81
Yang et al. (2018) [20]	Comparison	0.75–1.35 s	ipRGC activation: High > Low	t = 1.478

^†^ Recalculated using reported values

^1^ Only for male participants

Table 1 shows the relationship between color and temporal perception examined using various tasks of different durations; however, existing research displayed inconsistent findings. Smets et al. reported an overestimation of blue light in a verbal estimation and reproduction task. While the luminance of the lights was individually matched, the study did not account for potential changes in the saturation of different color stimuli [14]. Caldwell and Jones also conducted a temporal estimation task wherein participants estimated the passage of 30 s and 40 s in the presence of red, white, and blue lights. However, the results indicated that color had no consistent significant effects on temporal estimation [15].

Unlike earlier investigations using a tachistoscope, recent studies use displays for stimulus presentation, enabling control of the stimuli’s physical properties, such as brightness and saturation. Gorn et al. reported that temporal perception was shorter with a blue background than with red or yellow backgrounds; warm colors were perceived for a longer time than blue [16]. Shibasaki et al. reported that the perceived duration of red stimuli were perceived longer than that of blue stimuli. However, those results differed between the sexes; only men overestimated the duration of the red stimuli [18]. In contrast, Thönes et al. reported a temporal overestimation for blue stimuli but not for red stimuli [19].

The abovementioned studies show an inconsistency in the potential effects of color on temporal perception and indicate a lack of clarity regarding the probable mechanism of temporal perception modulation. Arousal is known to play an essential role in the distortion of perceived time, as suggested by the conspicuous internal-clock model (synonymous to the pacemaker–accumulator model) [12, 21, 22]. Indeed, studies that suggest that red stimuli can cause temporal overestimation, generally explain the mechanism by increased arousal. According to such models, arousal induced by the color red accelerates the internal clock (more time passes on the internal clock than in real life) or the speed of the pulse emitted per unit time (more pulses are stored in the accumulator), resulting in an overestimation of the duration of the red stimuli. Consequently, because the arousal induced by colors may primarily cause temporal modulation via its hue, the inconsistent results could be attributed to the challenge of controlling physical properties such as hue and luminance. In fact, physically brighter stimuli tend to be perceived longer (*e*.*g*., [23, 24]), and a recent study showed that perceptual brightness, which induces different pupillary responses, is sufficient to elongate the subjective time [25]. Hence, focusing on these inconsistencies may help us better understand the mechanism of temporal perception. The physical properties of the two colors, especially luminance and saturation, must be carefully matched and compared, as other physical aspects of color can affect individual arousal and subjective magnitude.

To overcome these limitations, we conducted a two-interval duration-discrimination task to assess the effect of equiluminant colors on temporal perception. Specifically, we colorimetrically controlled the saturation of the presented red/blue stimuli and matched the luminance with subjective equiluminant to segregate the former’s effect on temporal perception (Experiment 1). Additionally, since short-wavelength light (blue) is more effective than long-wavelength light (red) in evoking a pupillary light reflex (PLR) even at identical physical luminance (*e*.*g*., [26–28]), the second study evaluated the temporal distortion effect using pupil diameter-based equiluminant stimuli (Experiment 2). In fact, some recent studies have suggested an interesting connection between pupillary response and subjective passage of time (*e*.*g*., [29, 30]); however, few studies have focused on the effect of the PLR ([25]), which can be largely modulated by the physical properties of the stimuli to be timed. Therefore, with this study, we aimed to clarify the effect of color on temporal perception with controlled physical properties and to investigate the relationship between pupillary response, notably PLR and temporal perception.

## Experiment 1 (subjective equiluminance)

### Method

#### Participants

All experimental procedures and methods were approved by the Ethics Committee for Human Research at the Toyohashi University of Technology. Informed written consent was obtained after all procedural details were provided. The experiment involved 26 students (22 men, 4 women; age range: 21–27 years; M = 22.34, SD = 1.44) at the Toyohashi University of Technology. Eye movement data from two participants were excluded from pupillary analyses because the number of artifacts detected in the trials exceeded the 60% threshold, which could not be interpolated in the preprocessing phase. In the behavioral data analysis, trials with a reaction time > 10 s were also removed from the analysis, assuming poor task performance. This criterion rejected only a 0.18% of the trials. None of the participants reported color vision deficiency, and all had normal or corrected-to-normal vision.

#### Stimuli and apparatus

The experiment was conducted in a dimly lit room. MATLAB 2016a (The MathWorks, Natick, MA, USA) and the MATLAB toolbox, Psychtoolbox 3 [31–33], were used for stimulus presentation. The instructions and stimuli were presented on a liquid-crystal display (LCD) monitor (Display++, Cambridge Research Systems Ltd, Rochester, UK) with a resolution of 1,920 × 1,080 pixels and a refresh rate of 120 Hz. An eye-tracker (EyeLink 1000, SR Research, Oakland, Canada) was installed below the presentation display, centered on participants. The participant’s head was fixed on a chin rest at a viewing distance of 70 cm from the display. All behavioral responses were provided using a trackball or a numeric keypad with unnecessary keys removed.

In the temporal discrimination task (main task), we used two-color disk stimuli with a 10-degree visual angle diameter. We individually adjusted the luminance of the red stimulus in the adjustment task to match the subjective luminance of the blue stimuli, and saturation was controlled despite the difference in hues. In the adjustment task, we used a square stimulus (4 × 4 visual angle) instead. In both tasks, we displayed all experimental stimuli at the center of the screen.

The *xy* coordinates of the blue stimulus and the initial coordinates of red in the CIE1931 color space were (0.2286, 0.1936) and (0.4899, 0.3362), respectively. The background luminance remained constant at 37.83 cd/m^2^ for achromatic luminance (*x*, *y* = 0.3127, 0.329). During the stimulus presentation, the fixation cross was located at the center of the screen for pupil diameter recording.

#### Procedure

**Fig 1:**
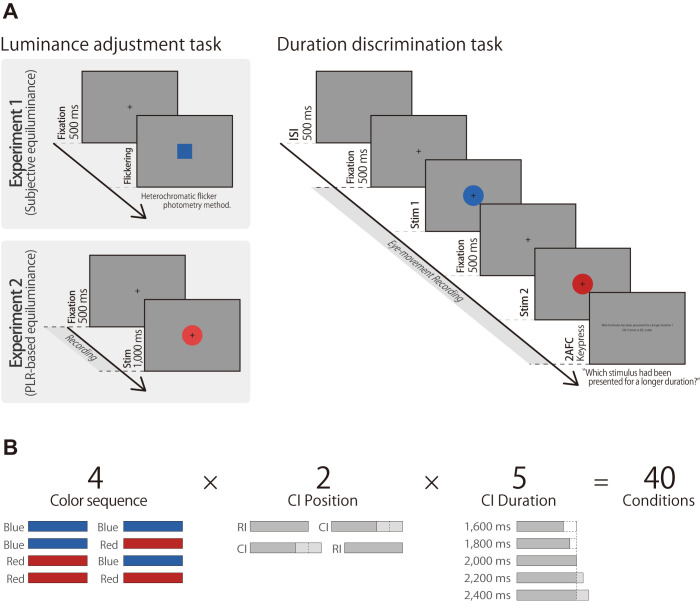
Experimental protocol for one trial. (A) Depiction of one trial sequence in the experiment. Stim1 and Stim2 in the duration-discrimination task refer to either the reference interval (RI) or comparison interval (CI). In Experiment 2, the luminance of the color stimuli was adjusted based on PLR amplitude instead of subjective equiluminance. (B) Illustration of experimental conditions for the duration-discrimination task.

The experiment was divided into two tasks: an adjustment task and a duration-discrimination task. The adjustment task consisted of eight trials based on the heterochromatic flicker photometry method [34]. The procedure of the duration-discrimination task was based on recent temporal perception studies [19, 25], with 320 trials conducted over four sessions. Fig 1 shows the protocol for one trial in each session and all the experimental conditions.

Each trial in the adjustment task (Fig 1A, left panel) began with the presentation of a fixation cross at the screen center followed by a flickering stimulus consisting of red and blue squares at the same location. The adjustments were made based on heterochromatic flicker photometry, a method for determining subjective equiluminance for a pair of heterochromatic stimuli in temporal alternation [34]. The flickering frequency of the two colors was set to 10 Hz, following previous studies [35], and the participants were instructed to adjust the intensity of the red stimulus until the flickering was mostly reduced (modulate to the null flickering point). Specifically, participants rotated the trackball upward or downward to increase or decrease the luminance of red (the Y-value), respectively, and pressed the button to select the displayed luminance. The initial Y-value of red was randomized over the eight trials, and the overall mean luminance was used in the duration-discrimination task.

Subsequently, the participants performed the two-interval duration-discrimination task (Fig 1A, right panel). Two-color stimuli were presented continuously on the screen followed by a 500-ms inter-stimulus interval (ISI). The red stimuli were individually generated by the Y-value determined in the adjustment task. The fixation point was displayed continuously while eye movement was recorded. In a single trial, one stimulus was presented for 2,000 ms (reference interval; RI), while the duration of the other stimulus varied between 1,600 ms and 2,400 ms in five steps (comparison interval; CI), separated by a 500-ms interval. Following continuous presentation, the participant indicated which visual stimulus was presented for a longer duration using the numeric keypad. The “4” key was used for the former stimulus, while “6” was used for the latter. No feedback was provided. The order of CI/RI positions was randomized and counterbalanced across sessions following a within-subjects design; the three factors were fully cross (color sequence, CI position, and CI duration). Each of the 4 (color sequence) × 2 (CI position) × 5 (CI duration) = 40 combinations were presented for 8 times, resulting in a total of 320 trials per participant (Fig 1B).

The eye tracker was adjusted before the first and the subsequent sessions, as necessary, using a standard five-point calibration. Participants were neither instructed to nor discouraged in performing temporal-judgment strategies, such as counting up and other rhythmic activities known to increase temporal sensitivity [36].

### Data analysis

#### Eye movement measurement data

An eye tracker (EyeLink 1000, SR Research, Oakland, Canada) recorded binocular pupillary responses at a sampling rate of 500 Hz. We did not instruct the participants when to blink during eye movement measurements since we placed importance on the temporal task; hence, we interpolated blinks before the main analysis using cubic-spline interpolation [37] in MATLAB 2018b. Pupil size was expressed in arbitrary diameter units (EyeLink values), which were generated by the eye tracker. In the time-course analysis, pupil diameter data were baseline-corrected by subtracting the mean pupil size during −50 ms to 0 ms (stimulus onset), and filtered using a 20-point moving average.

#### Behavioral data

The participants reported which stimulus had a longer perceived duration, either RI (fixed duration of 2,000 ms) or CI (1,600–2,400 ms in 200-ms steps), in the two-interval duration-discrimination task. To estimate the psychometric functions, the participants’ responses (proportion of CI stimuli judged longer) were modeled by fitting the logistic psychometric function using the Palamedes toolbox in MATLAB [38] and computing the point of subjective equality (PSE) and just-noticeable difference (JND). The “threshold” and “slope” parameters used in the toolbox function were left at their default free parameters for all color sequence conditions (B–B, B–R, R–B, and R–R; “B” and “R” for blue and red, respectively, representing the CI–RI combination). Since the PSE represented the level of duration continuum which is subjectively identical to the duration of the RI, the PSE was used to compare the effect of hue on temporal perception. The statistical analyses were performed using R for macOS version 3.5.1 and an repeated-measures analysis of variance (ANOVA) function, anovakun (version 4.8.2). Pairwise comparisons of the main effects were corrected for multiple comparisons using Shaffer’s Modified Sequentially Rejective Bonferroni (MSRB) procedure. In the ANOVA, the partial *η* ($\eta_{p}^{2}$) was reported as a measure of association strength (effect size). In addition to the analysis in R, JASP [39] was used to compute the Bayes factor BF_10_ in a Bayesian repeated-measures ANOVA, and BF_01_ as relative evidence to interpret the effect of the null hypothesis.

### Results

#### Adjustment task

In the adjustment task, the participants conducted a luminance-matching task based on heterochromatic flicker photometry to determine subjectively equiluminant Y-value for the red stimulus. The mean average of the adjusted Y-value was 0.308, almost identical to the Y-value of blue (0.300). Therefore, we conducted a one-sample Bayesian t-test with a test value of 0.300 on the adjusted Y-values to statistically support this hypothesis. The analysis indicated a Bayes factor BF_10_ of 0.427 and a BF_01_ of 2.34, which can be interpreted as anecdotal evidence to support H0 over H1 [40]. The mean average of the adjusted Y-value is statistically identical.

#### Duration judgment

**Fig 2:**
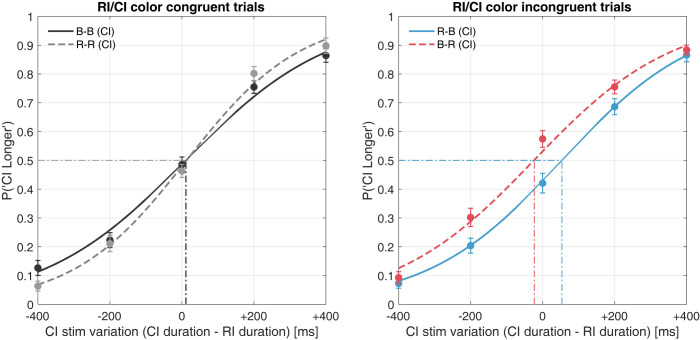
Mean psychometric functions (subjective equiluminance). Left panel: Psychometric function fitting by the proportion of comparison interval–longer (CI) response in congruent color sequence. Right: For an incongruent color sequence. Abbreviations: B–blue, R–red, CI–comparison interval, RI–reference interval.

In the duration-discrimination task, the participants indicated whether the RI (duration fixed at 2,000 ms) or CI (variable duration: 1,600–2,400 ms in steps of 200 ms) stimulus was perceived to have a longer duration. The order of the presented CI was not predetermined, and the CI was presented first (CI position: CI–RI) or second (RI–CI) with equal probability. Each CI-longer probability was used for psychometric function fitting for each duration step of the CI to compute the PSE and JND. The mean psychometric function shown in Fig 2 was derived by fitting a psychometric function to the average of the five duration steps of the CI. The difference in duration was computed by subtracting the RI duration from the CI duration.

**Fig 3:**
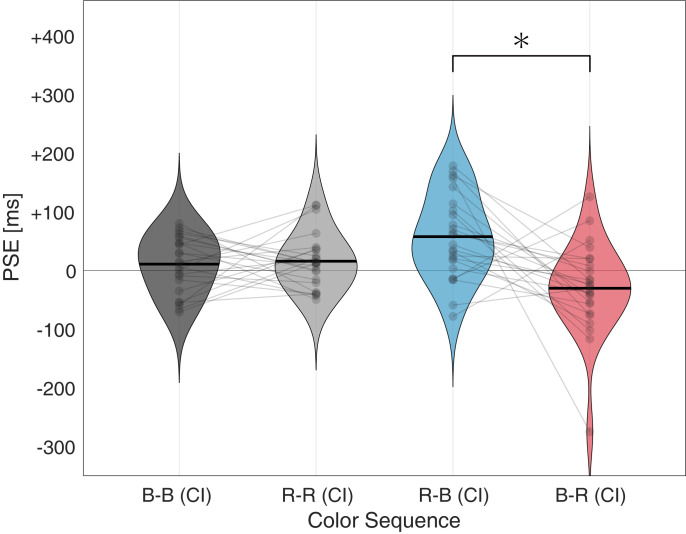
Mean PSE of each color sequence. Violin plots of mean PSE as a function of stimulus sequence. The plots show the data distribution with a solid black line for the mean of the data. The overlaid semitransparent dot indicates the mean value for each participant’s data. * Indicates statistically significant (*p* < 0.05) differences in the analysis of variance and post-hoc testing. Abbreviations: B–blue, R–red, CI–comparison interval, PSE–point of subjective equality.

Each solid line in Fig 2 represents a fitted psychometric function. The color conditions of the RI and CI stimuli are indicated via the labels (*e*.*g*., R–B represents the RI stimulus being red and the CI stimulus being blue). The dashed vertical lines represent the PSEs under each condition, proportional to the 50% probability of the function; the PSE denotes a specific duration of the CI judged equivalent to the RI, which is presented for 2,000 ms. A relative shift in the fitted function was observed in the incongruent stimulus comparison, whereas no shift emerged in the congruent stimulus comparison. Fig 3 shows the PSE determined using a psychometric function.

A one-way repeated-measures ANOVA was performed on the color sequence, the main effect of which was significant (F[3, 69) = 6.5613, *p* = 0.0006, $\eta_{p}^{2}$ = 0.2060) in the PSE; therefore, a post-hoc *t*-test was also conducted. The post-hoc analysis showed that the two incongruent color sequences, R–B and B–R, differed significantly, as the PSEs were smaller when the CI color was red rather than blue (*t*[23] = 3.4053, adjusted *p* = 0.0146), whereas no other color sequence combination showed significant differences. Additionally, the Bayesian repeated-measures ANOVA indicated BF_10_ = 36.5 and BF_10_ = 1.027 for PSE and JND, respectively. The PSE shift suggests a temporal overestimation of red stimuli compared with blue stimuli. Contrary to the PSE, the effect of color sequence on JND did not reach statistical significance (F[3, 69] = 2.7088, *p* = 0.0518, $\eta_{p}^{2}$ = 0.1054), suggesting that temporal sensitivity did not differ by color sequence.

#### Pupillary response

**Fig 4:**
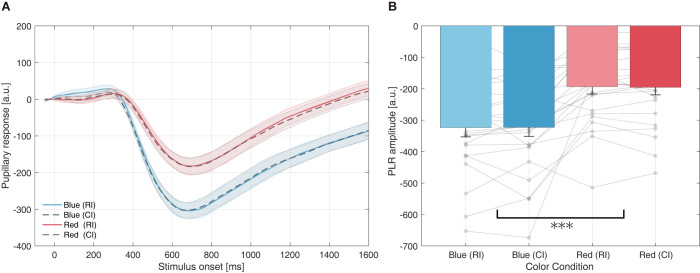
Pupillary response to each color stimulus (subjective equiluminance). (A) Mean change in pupil diameter from stimulus onset. Each line represents pupillary responses to RI and CI stimuli, whereas the shaded regions are standard errors of the mean. The x-axis range is limited to 1,600 ms, the shortest CI duration. (B) Mean peak PLR amplitude of each color condition. Peak PLR amplitude is determined as the minimum pupil diameter between 300–1,300 ms in the time domain. *** Indicates a statistically significant (*p* < 0.001) main effect by ANOVA. The semitransparent dots indicate each participant’s data. Abbreviations: ANOVA–analysis of variance, CI–comparison interval, PLR–pupillary light reflex, RI–reference interval.

All pupillary responses to the red and blue stimuli in RI and CI durations were averaged across conditions after the raw pupil data were preprocessed, as explained in the Methods section. Fig 4A shows the grand average of pupil responses during a −50-ms to 1,600-ms stimulus onset under both color stimuli and presentation (RI or CI) conditions. The range was set to the shortest CI duration and the responses were aligned according to the presentation onset.

As shown in Fig 4A, a typical PLR was observed for each color stimulus. Thus, the peak PLR amplitude was computed using the average minimum pupil diameter between the 300–1,300 ms time domains, as depicted in Fig 4B. This figure shows that the two-way repeated-measures ANOVA (color condition × stimuli-type condition) on the effect of peak PLR amplitude revealed a significant main effect of color condition (*F*[1, 23] = 31.7220, *p* < 0.001, $\eta_{p}^{2}$ = 0.5797). Post-hoc comparisons also suggested a significant difference between the color conditions in each stimulus-type condition. However, the stimulus-type condition, did not reach statistical significance (*F*[1, 23] = 0.0059, *p* = 0.9393, $\eta_{p}^{2}$ = 0.0003). These results suggest that blue evoked a larger PLR than red, despite the two colors being individually matched based on subjective equiluminance.

### Discussion

In the first study, we investigated whether the perceived duration of a visual stimulus was affected by hue in subjective equiluminant conditions. This was also conducted to examine previous studies’ results that contradict each other (*e*.*g*., [18–20]), under stringent control of physical properties, since these may be due to lack of control of the physical properties, especially the luminance of red and blue stimuli. Furthermore, we performed pupillometry to investigate the relationship between the PLR response and temporal perception, as a recent study suggested possible associations between the two [25], with the former differing under stimuli of different wavelengths [26]. Based on the known effects of arousal on temporal perception and the vital function of red, we hypothesized that the participants would overestimate (perceive longer) the duration of red relative to blue.

The participants performed a two-interval duration-discrimination task at supra-second range intervals. According to the psychometric function analysis, the shift in the fitted function indicated that the duration of red stimuli was perceived as longer than that of blue stimuli (Fig 2, right panel). The mean difference in the PSE suggested that the durations of the blue and red stimuli were perceived as identical when the red stimulus was actually displayed 88 ms shorter (4.28%) than the blue stimulus (Fig 3).

Moreover, we must emphasize that this effect was observed even when the colors of the two stimuli were controlled for saturation (colorimetric adjustments in terms of the CIE1931 color space) and luminance differences (individually adjusted to subjective equiluminance using the heterochromatic flicker photometry method). Our results suggest that the temporal distortion effect of red and blue is primarily driven by hue and that the effects of luminance and saturation are partially limited. Moreover, notwithstanding the adoption of the heterochromatic flicker photometry method to satisfy subjective equiluminance conditions, the mean average of the adjusted Y-value was almost identical, and the red and blue stimuli were also closely physically equiluminant in mean.

Furthermore, when we examined the pupillary responses, the PLR profile differed considerably, especially in amplitude. At the PLR peak for the blue stimulus, the pupil diameter was 1.66 times larger than that for the red stimulus (Fig 4). Since the PLR is driven by known photoreceptors, such as rods, cones, and intrinsically photosensitive retinal ganglion cells (ipRGCs) [41], all of which have different sensitivity characteristics [42], the amplitude of the PLR to red/blue stimuli is known to differ [43]. Szabadi et al. reported that short-wavelength light is more effective than isoluminant long-wavelength light in evoking the PLR because of the stimulation of melanopsin-containing photoreceptors [26]. Our pupillary response data also showed that the PLR amplitude differed according to hue under subjective equilibration.

Bombeke et al. reported that differences in pupil diameter could modulate the magnitude of the initial feedforward response in the primary visual cortex and could, therefore, represent a confounding variable in studies investigating the neural influence of psychological factors [44]. Suzuki et al. also discovered that stimuli that induce larger constrictions causally reduce the amount of light emitted to the retina, causing a drop in EEG signals, specifically steady-state visual evoked potentials (SSVEPs; an EEG that reflects the attention level to visual stimuli, typically recorded from occipital channels) recorded via electrodes located in the occipital area [45]. Since the apparent duration is typically known as a positive function of sensory signal intensity, various factors leading to a larger PLR in response to blue stimuli may explain the effect of color on temporal perception based on sensory signals. In conclusion, our findings suggest that red stimuli were temporally overestimated compared to blue stimuli under subjective (nearly physical) equiluminance. However, as the PLR differs according to the hue of the stimulus, its effect on temporal perception must be clarified to fully elucidate the potential effect of color among inconsistent research findings. Hence, to determine the relationship between temporal perception, hue, and pupillary responses, we conducted an additional control study with equiluminant stimuli based on PLR-matched luminance.

## Experiment 2 (PLR-based equiluminance)

### Method

#### Participants

As with Experiment 1, all procedures were approved by the Committee for Human Research at the Toyohashi University of Technology. Informed written consent was obtained after all procedural details were explained. Sixteen students (11 men, 5 women; age range: 20–34 years; M = 25.69, SD = 3.65) at the University of Oslo took part in the experiment. Two participants’ eye movement data were excluded from pupil analyses, and 2% of the trials were rejected based on excessive reaction time. The rejection criteria for both eye movement data and behavioral data were identical to those in Experiment 1.

#### Stimuli and apparatus

The task was conducted in a different dimly lit room using MATLAB 2019a (The MathWorks, Natick, MA, USA) and MATLAB’s Psychtoolbox, as mentioned in Experiment 1. The experimental stimuli were presented on an LCD monitor (P2213, Dell Inc.) with a resolution of 1,680 × 1,050 pixels and a refresh rate of 60 Hz. Colorimetric calibrations were conducted in advance for linear light output, and maximum luminance was corrected to 120 cd/m^2^ to match the display specifications in Experiment 1. The apparatus arrangements also followed those in Experiment 1, except that the viewing distance was set to 66 cm from the screen. All behavioral responses were delivered via a numeric keypad with unnecessary keys removed.

The chromatic properties of the stimuli presented in the temporal discrimination task (main task) were identical to those in Experiment 1. However, the luminance of the stimuli was computed individually in the PLR-matching task. In this task, the luminance of the colored disks (diameter of 10-degree visual angle) varied at Y = 0.22, 0.25, 0.28, 0.31, and 0.34.

#### Procedure

The procedure of the temporal discrimination task was nearly identical to that in Experiment 1. However, instead of the heterochromatic flicker photometry method, a PLR-based method was used in Experiment 2 (Fig 1A, left panel).

In the PLR-matching task, each trial began with a presentation of the fixation cross for 500 ms followed by a red/blue disk for 1,000 ms at the center of the screen. The participants were instructed to passively view the stimuli. The two factors were fully crossed (2 [color] × 5 [luminance) = 10 combinations) and randomly presented in a total of 50 trials per participant over two sessions. After stimulus presentation, the pupillary response was analyzed offline to determine a specific Y-value of blue. After preprocessing the pupillary data, the average PLR peak of all ten conditions was computed, and a linear regression analysis was performed to fit the discrete data points of luminance and PLR amplitude for both red and blue. Finally, luminance was evaluated via a fitted polynomial at the point where the PLR amplitude of red became equivalent. Simply put, the Y-value of blue that matched the PLR amplitude of red (Y = 0.300), was calculated. This calculated value was used to generate the blue stimulus in the following duration-discrimination task.

Followed by a sufficient break, the participants performed the two-interval duration-discrimination task (Fig 1A, right panel). As mentioned above, the procedure of the main task was identical to that of Experiment 1. Fig 1B depicts the combination of the 40 experimental conditions.

### Data analysis

#### Eye movement measurement data

All aspects of behavioral data analysis were analogous to those in Experiment 1, whereas pupil size and eye movements were measured using the SensoMotoric-Instruments RED500 (SMI, Berlin, Germany) eye-tracking system at a sampling rate of 250 Hz. A nine-point calibration was performed before each session in the PLR-matching and duration-discrimination tasks. In both phases, participants were not instructed when to blink, and, thus, blinks and artifacts were interpolated before analysis using the same method as in Experiment 1 [37]. Trials that retained additional artifacts, computed by thresholding peak changes in the velocity of the pupillary response, were excluded from analysis.

Pupil size was measured in SI units (mm) by the eye tracker. Because of the specifications of the eye-tracking system, the units varied from those in Experiment 1. Measurements in terms of absolute units may not be entirely invariant to factors such as viewing distance and are, thus, somewhat arbitrary [46]. Therefore, for all time-course analyses conducted in this study we used baseline-corrected relative values, which consider random fluctuations in pupil size over time and allowed us to compare results in different units. As for Experiment 1, pupil diameter data were filtered using a 20-point moving average.

### Results

#### PLR-matching task

**Fig 5:**
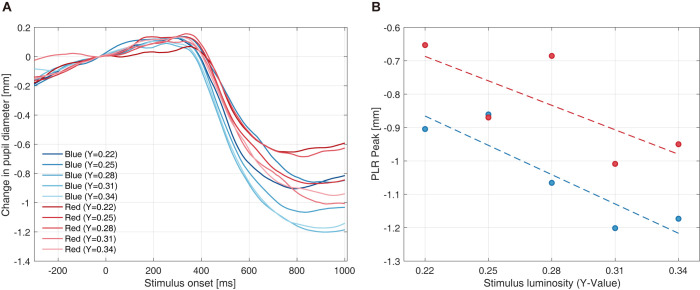
Example pupillary response and peak PLR amplitude. (A) One participant’s mean pupillary response to different hues and luminances (color and luminance: two (color) × five (luminance) = ten combinations). Solid lines represent pupillary responses for each condition. (B) An example of mean peak PLR amplitude for each color condition. PLR diameter is computed as the minimum pupil diameter. The two dashed lines represent the fitted linear regression model, composed of discrete luminance and peak PLR amplitude data points, for red and blue stimuli, respectively. Abbreviations: PLR–pupillary light response.

Raw pupil data were preprocessed, as explained in the Methods section. Fig 5A shows the pupillary response for one participant.

The typical PLR profile was observed from the responses in hue and luminance conditions; that is, a tendency for (1) PLR amplitude (constriction amount) to increase with luminance and (2) blue to induce a prominent PLR. The first finding can be explained by pupil constriction in response to brightness, a function of retinal illuminance control of physical inputs from the ambient environment [42]. Second, as short-wavelength light is more effective than long-wavelength in evoking the PLR, pupil constriction was larger for the blue stimuli. Therefore, we computed the average of the minimum pupil diameter between the 400–1,000 ms time domains in each condition, as shown in Fig 5B, with discrete data points of luminance and PLR peaks. Although Watson and Yellott reported a unified formula wherein luminance and pupil diameter had a logarithmic relationship over a wide range of luminances (10^−4^ to 10^4^ cd/m^2^) [47], the intensity of the stimuli used in this study was limited to a modestly narrow luminance range (*e*.*g*., approximately 26.4–39.6 cd/m^2^ in achromatic luminance). Hence, we concluded that linear regression provided adequate fitting accuracy, as indicated by the dashed lines in Fig 5B. The mean calculated Y-value was 0.3037 (SD = 0.0586).

#### Pupillary response

**Fig 6:**
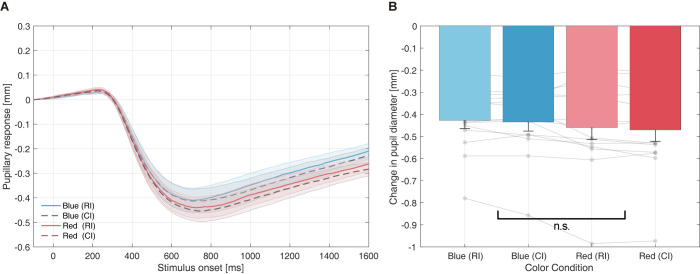
Pupillary response to each color stimulus (PLR-based equiluminance). (A) Mean change in pupil diameter from stimulus onset. Each line represents pupillary responses to RI and CI stimuli, whereas the shaded regions are standard mean errors. (B) Mean peak PLR amplitudes for each color condition. Peak PLR amplitude is determined as the minimum pupil diameter between 300–1,300 ms in the time domain. The overlaid semitransparent dots indicate each participant’s data. Abbreviations: CI–comparison interval, PLR–pupillary light reflex, RI–reference interval.

As previously conducted, we tracked pupil diameter changes induced by the presentation of color stimuli in CI positions and all CI durations. Fig 6A shows the grand average of the pupil response between 50 ms before and 1,600 ms after stimulus presentation under each color and stimulus-type condition. Furthermore, the profile of a typical orienting response (approximately 200 ms from onset) [48] and the PLR were observed (approximately 300–700 ms from onset). Fig 6B shows the minimum mean pupil diameter of the PLR, computed by the average of the minimum peak PLR diameter between the 300–1,300 ms time domains.

Two-way repeated-measures ANOVA (color condition × stimulus-type condition) on the effect of peak PLR amplitude showed no significant main effect on color condition (*F*[1, 13] = 2.5119, *p* = 0.1370, $\eta_{p}^{2}$ = 0.1619) or stimulus-type condition (*F*[1, 13] = 2.9737, *p* = 0.1083, $\eta_{p}^{2}$ = 0.1862). These statistical findings support the fact that PLR matching was performed.

#### Duration judgment

**Fig 7:**
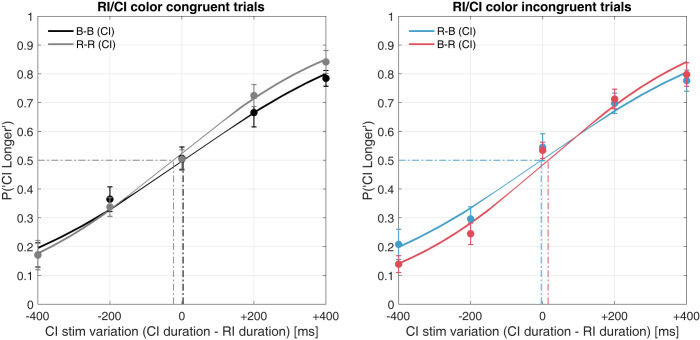
Mean psychometric functions (PLR-based equiluminance). Left panel: Psychometric function fitting according to the proportion of comparison interval–longer (CI) responses in congruent color sequence. Right: For incongruent color sequence. Abbreviations: B–blue, R–red, CI–comparison interval, RI–reference interval.

The CI-longer probabilities for all CI durations were again used for psychometric function fitting. The PSEs for each color sequence condition (B–B, R–R, R–B, and B–R) were calculated by fitting a cumulative normal function and determining the 50% point. Fig 7 shows the mean psychometric function computed using the average of all participants’ CI-longer proportions in each CI duration condition.

From the estimated psychometric function, no significant shift was observed in congruent- or incongruent stimulus comparisons. The computed PSEs and JND were used for statistical testing. A one-way repeated-measures ANOVA was conducted on each color sequence; the effect for the PSE was not significant (F[3, 39] = 1.1105, *p* = 0.3565, $\eta_{p}^{2}$ = 0.0616), and the effect size was relatively small [49]. In contrast, a significant main effect was confirmed for the JND (F[3, 39] = 3.1195, *p* = 0.0369, $\eta_{p}^{2}$ = 0.1935); however, none of the pairs in the post-hoc comparisons reached statistical significance. Bayesian repeated-measures ANOVA indicated BF_10_ = 3.012 and BF_10_ = 1.718 for the PSE and JND, respectively. The Bayes factor of the JND analysis (BF_10_ = 1.718, BF_01_ = 0.582) reaching statistical significance in the classical ANOVA, indicated anecdotal evidence to support H1 over H0 [40, 50]. Notably, the statistics suggested no shift in the PSE—in other words, no significant temporal distortion due to the colors.

### Discussion

To clarify the causal role of PLR amplitude induced by the to-be-timed stimuli and temporal perception, we replicated the first experiment in the second control experiment, excluding the equiluminance method, to investigate whether the perceived duration of the visual stimulus is affected by hue in PLR-matched conditions. This objective was based on two study backgrounds: (1) previous studies investigating the temporal distortion effect on hue show inconsistent results, which may be due to the failure of stimulus intensity matching; and (2) pupil diameter differs depending on the hue, which can play a role in temporal distortions, in addition to the arousal level induced by the hue. To the best of our knowledge, this study is the first attempt to investigate the relationship between the PLR response, temporal perception, and stimulus color.

Regarding PLR matching, Fig 6 illustrates that the PLR profile and peak constriction were similar among the hue conditions. As PLR amplitude generally differs in equiluminant color conditions because of stimulation of different photoreceptors, the results suggest adequate individual PLR matching in the first task. Specifically, the mean Y-value applied for the blue stimuli was 0.284, smaller than that for red (0.300). Since blue light is more effective in evoking the PLR, the actual value is expected to be lower than the computed value.

Statistical analysis suggested no significant temporal distortion via the hue condition, even though arousing stimuli are typically reported to be perceived longer. Many researchers have reported that observing emotional expressions can cause temporal distortion by increasing perceivers’ arousal levels (*e*.*g*., [51]). Moreover, because red is considered a color of arousal, it can induce time overestimation. However, interestingly, the mean luminance to match the PLR amplitude was smaller for blue than for red, in this study. By comparing the physical luminance of the stimuli in Experiment 1, blue was adjusted to be darker in this study, whereas red was almost identical (Y-values of 0.300 and 0.284, respectively). Considering that “apparent duration is typically a positive function of the sensory signal intensity,” red should have been perceived even longer, as the luminance of the comparative blue was adjusted to be smaller. In conclusion, our findings suggest that the temporal distortion effect of hue does not occur under PLR-based equiluminance, and it may be plausible that stimulus intensity, especially the physical luminance and arousal, is not the sole driving factor of temporal distortion.

## General discussion

This study investigated whether the color of a visual stimulus affects its perceived duration in different equiluminant conditions, to uniformly explain inconsistent research findings (see Table 1 for the results of previous temporal perception studies).

In Experiment 1, the red/blue stimuli were matched by subjective luminance using heterochromatic flicker photometry. From the behavioral results, a shift in the PSE was observed in incongruent color conditions, indicating an overestimation of red stimuli compared to blue stimuli. In contrast, our analysis suggests no JND modulation in temporal judgments. These behavioral results aligned with those of previous studies (*e*.*g*., [14, 16, 18]) that observing emotional expressions involving the color red can lead to temporal distortion. Hence the overestimation of the duration of red stimuli in Experiment 1 may be due to the hue-induced arousal effect. Notably, this effect was observed even when the colors of the two stimuli were controlled for saturation (colorimetric adjustments in the CIE1931 color space) and subjective luminance; consequently, the adjusted luminance was also physically equiluminant. However, continuous pupillary recordings revealed significant differences in the PLR between the two stimuli. The blue stimulus induced notable constriction relative to the red stimulus. Several researchers have reported the possibility of differences in pupil diameter to directly modulate the magnitude of the response in the primary visual cortex, and stimuli that induce larger constrictions can causally reduce the amount of light entering the retina, leading to a drop in EEG amplitude in the primary visual cortex [44, 45]. Moreover, because apparent duration is typically a positive function of sensory signal intensity, pupillary responses such as the PLR should also be considered alongside physical aspects as factors that modulates the subjective passage of time. Therefore, we conducted a control experiment that adapted the duration-discrimination task to PLR-based equiluminant conditions (Experiment 2). Psychometric functional analysis in Experiment 2 revealed no significant distortion of duration according to hue, when the PLR was individually matched; the overestimation of the duration of the red stimulus faded when the stimulus luminance was matched based on the PLR amplitude. An additional two-way repeated-measures ANOVA on the effect of the PSE, with color sequence as a within-subjects factor and experiment condition as a between-subjects factor, was conducted to support the notion that the PSE effect significantly differs when the PLR is not controlled (Experiment 1) vs. when it is controlled (Experiment 2). Note that these analyses were conducted on data with unequal sample sizes due to different participant numbers in the experiments. The analysis revealed a significant main effect on experimental condition (F[1, 36] = 6.205, *p* = 0.017, $\eta_{p}^{2}$ = 0.147), and on the interaction of color sequence and experimental condition (F[3, 108] = 4.807, *p* = 0.004, $\eta_{p}^{2}$ = 0.118). Post-hoc comparisons on the interaction, showed a significant difference in three color sequence pairs on the effect of the PSE, (Exp2-RR–Exp1-RB [*t* = -3.653, adjusted *p* = 0.010], Exp1-RB–Exp2-RB [*t* = 3.383, adjusted *p* = 0.024], and Exp1-RB–Exp2-BR [*t* = 3.434, adjusted *p* = 0.023]). This interaction supports the fact that different equiluminance methods can result in differences in temporal perception.

Although the physiological models suggesting a differential effect of different PLR amplitudes on temporal perception are currently lacking, our newly found causality between the equiluminance method and temporal perception can be explained by neural intensity fluctuations based on the amount of incident light entering the pupil. The PLR generally controls pupil diameter to assist in vision adaptation via pupil constriction against intense ambient luminance and dilation in a dark environment. Therefore, the physical constriction of the pupil reduces the number and probability of photons captured by the retina. Notably, recent studies suggest that the PLR is not merely a reflex to brightness, but also affected by higher-order factors [42]. As a matter of fact, pupil size also fluctuates according to hue (*e*.*g*., [26–28, 43]), and even illusory brightness and subjectively perceived luminance (*e*.*g*., [52–54]). Furthermore, Kinzuka et al. recently reported that temporal perception is also modulated by the illusory brightness of a glare illusion (a visual illusion that enhances perceived brightness without changing physical luminance) [25], resulting in a different PLR response during the to-be-timed stimuli presentation [55, 56]. In this study, the authors explained this probable temporal distortion mechanism as an internal magnitude decrease in the illusory brighter stimuli caused by the reduced light entering the eye from pupillary constriction. Recently, Suzuki et al. reported that the amplitude of SSVEPs decreased in accordance with constriction-eliciting stimuli [45]. Moreover, they described this inhibition of SSVEPs as a causal relationship between illusion-induced pupil constriction and less light entering the pupil. These findings agree that the duration of red stimuli (relatively larger pupil diameter) was considered longer than blue (relatively smaller pupil diameter); the energy entering the eye was higher when the luminance of the stimuli was controlled by subjective (≈ physical) equiluminance (Experiment 1). In fact, the pupil diameter at the PLR peak induced by the red stimulus was only approximately 60% of that induced by the blue stimulus.

Furthermore, Katsuura et al., who examined the effect of red and blue colors on temporal perception, reported that the cortical activity level in the visual pathway (retina-lateral geniculate body-primary visual cortex) might influence the temporal modulation effect, as mentioned earlier [17]. Since other researchers noted that pupil constriction and less light entering the pupil can result in decreased activation in visual-related brain regions, this may cause temporal underestimation of the interval, an assumption based on the coding efficiency model [57]. In this model, Eagleman and Pariyadath proposed that neural coding efficiency and the degree of activation provide a basis for temporal perception. Several subsequent studies revealed that various non-temporal factors that lead to temporal overestimation also evoke larger neural responses (*e*.*g*., [12, 58]). Despite the speculative relationship between the cortical activity level in the visual pathway and temporal perception, from these relations, the disappearance of temporal modulation can be explained by the indirect control of the internal magnitude of the to-be-timed stimuli, by matching the PLR. In Experiment 1, the stimulus luminance was matched based on subjective and physical luminance. As is evident from Fig 4, PLR constriction was weaker for the red stimulus; that is, the neural activity in the visual pathway of red stimuli was considered larger than that of blue stimuli. According to the coding efficiency model, stimuli that induce more extensive neural activity are perceived to be displayed for a longer period of time; hence, the local activity evoked by the red stimulus can explain why it was perceived to be displayed for a significantly longer period than the blue stimulus. However, in the second control experiment, because the luminances of the two colors were individually controlled to match the peak PLR amplitude, indirectly controlling the photons captured by the retina, it is possible that the temporal modulation may not have occurred. Although we cannot discount the hue-arousing effect on temporal distortion since we were not able to directly assess subjective arousal, the results from both experiments suggest another possible mechanism of how the hue of a visual stimulus can modulate temporal perception. The inconsistent results noted in Table 1 could be attributed to the challenge of controlling physical properties, which results in different PLR amplitudes.

Here, we will note few limitations to the study. First, temporal processing mechanisms may vary between sub-second (milliseconds) and supra-second (seconds to minutes) interval ranges [59, 60]. Additionally, recent studies have suggested that the PLR profile differs in late components in response to a blue stimulus, as ipRGCs (which respond to blueish light) [41] respond more slowly to light than rods and cones do. As this study was focused on the judgment of display intervals ranging from 1,600–2,400 ms, the color effect and the implications of the PLR on temporal perception may differ in other temporal ranges. Second, the experimental environment may not have been stringently controlled because the two studies were conducted in different locations on different participant groups. Despite colorimetric calibration and baseline correction, we cannot entirely exclude the possibility that the luminance of the display or either room was not completely identical. As baseline pupil diameter constitutes various factors, some aspects, such as cognitive load (*e*.*g*., difficulties in the duration-discrimination task) and pupil near response (*e*.*g*., visual distance) might affect the pupil diameter during the task [42]. These effects are considered negligible since we compared pupillary responses within each study and focused solely on the relative changes in pupil diameter. However, in future studies, eye-tracker models and experimental environments, including the participant group, should be controlled, to analyze the pupil diameter baseline and directly compare the results within experiments.

In our analyses of psychometric functions and pupil diameter, our results showed that the perceived duration of red was overestimated compared to blue, even when the physical aspects of the stimuli were strictly controlled in subjectively (≈ physically) equiluminant conditions. However, this overestimation faded when the luminances of the two stimuli were matched based on the PLR amplitude. This causality between the equiluminance method and temporal perception may be due to the amount of incident light entering the pupil, leading to the modulation of different degrees of neural activation in visual-related regions. These results suggest another possible mechanism of temporal distortion by hue, in addition to the previously discussed hue-arousing effect. In addition, our results suggests that the pupillary somehow plays a role in temporal perception from a phenomenological perspective. Although these links remain speculative at present and require further consideration, incorporating neuroscientific approaches and discussion on theoretical frameworks, we believe that discovering this causal role may help clarify the inconsistent results of previous studies and further elucidate the underlying mechanisms of temporal perception.
